# The Adoption of QR Code Mobile Payment Technology During COVID-19: A Social Learning Perspective

**DOI:** 10.3389/fpsyg.2021.798199

**Published:** 2022-02-16

**Authors:** Ming Tu, Lei Wu, Hua Wan, Zhoujin Ding, Zizheng Guo, Jiayi Chen

**Affiliations:** ^1^College of Economics and Management, Huazhong Agricultural University, Wuhan, China; ^2^Business College, Shaoxing University, Shaoxing, China; ^3^School of Business Administration, Zhongnan University of Economics and Law, Wuhan, China; ^4^Macau University of Science and Technology, Macao, Macao SAR, China

**Keywords:** QR code mobile payment, COVID-19, social learning theory, perceived severity, social influence, utilitarian benefit, health benefit

## Abstract

The increasing number of quick response (QR) code mobile payment users heralds the coming of a cashless society. However, the extent to which the coronavirus disease 2019 (COVID-19) pandemic accelerated the adoption of QR code mobile payment has not been sufficiently researched. Based on social learning theory, this study models how external interaction with the environment has affected the internal appraisal and behavioral intention to adopt QR code mobile payment during COVID-19. Empirical results from 248 respondents revealed that perceived severity and social influence positively affected the perception of utilitarian and health benefits of respondents, which in turn influenced the behavioral intention to use the QR code mobile payment. The theoretical contribution and managerial implications of this study are also discussed.

## Introduction

A growing number of consumers prefer to make payments *via* quick response (QR) codes scanned with their mobile phones over using cash or bank cards in physical stores. According to a survey published by the China Internet Network Information Center (CNNIC) in February 2021, 8.74 million new mobile payment users were added between March and December 2020 (CNNIC, 2021). A large-scale survey conducted by China UnionPay revealed that QR code payments represented 85% of all mobile payments in 2020 (CUP, 2021). The popularity of QR code payments in physical stores heralds a cashless future.

Existing literature on mobile payment heavily emphasizes payment system characteristics (e.g., mobility, security, and convenience) and user-centric factors (e.g., innovativeness and optimism) (Kim et al., 2010). However, less is known about the factors that contributed to the rapid growth in popularity of QR code mobile payment in 2020; in particular, it is unclear whether the external environmental stimulus of the COVID-19 pandemic accelerated the adoption of this payment system. For example, scholars have called for closer attention to the health benefits of using QR codes during the COVID-19 pandemic (Yan et al., 2021). Therefore, it is desirable to better understand the determinants of QR code mobile payment adoption in the context of COVID-19.

This study aims to examine the increasing use of QR code mobile payment from a social learning theory perspective by considering the impacts of both external environment interaction and internal psychological appraisal. Specifically, this study addresses the following questions: (1) How does internal psychological appraisal (e.g., utilitarian benefit and health benefit) affect the adoption of QR code mobile payment? (2) What are the effects of the perceived severity of the COVID-19 pandemic on the internal psychological appraisal of QR code mobile payment of consumers? Did the pandemic further motivate the adoption of this form of payment?

This study makes three significant contributions. First, while previous studies have focused mainly on utilitarian factors (de Kerviler et al., 2016; Park et al., 2019; Yan et al., 2021), this study adds new knowledge to the literature on QR code mobile payment by emphasizing the appraisal of health benefits in the adoption process. Second, unlike prior research that has considered mobile payment merely as a transaction tool in the common consumption context (Teo et al., 2015), this study enriches the literature on mobile payment by investigating the adoption of QR code mobile payment of consumers in response to the threat of COVID-19. Finally, this study examines the interaction between the external environment and the internal psychological appraisal of consumers to investigate the process of adoption of QR code mobile payment by applying and validating social learning theory in the mobile payment context. Overall, this study not only provides a theoretical assessment of the adoption of QR code mobile payment during the COVID-19 pandemic but also has managerial implications for companies wishing to expand QR code mobile payment to respond to disease outbreaks.

In the remainder of this study, we first reviewed prior literature on mobile payment adoption. Then, we applied social learning theory to mobile payment adoption behaviors. Then, we elaborated on how people carry out the external interaction and internal psychological processes that drive them to use mobile payment. Finally, we presented a questionnaire-based survey and a partial least squares structural equation modeling (PLS-SEM) method to empirically confirm our prediction of QR code mobile payment usage.

## Theoretical Background

### Mobile Payment

Mobile payment is defined as the use of a mobile device to complete an economic transaction (Liébana-Cabanillas et al., 2014). Mobile payment enables the purchase of either digital goods (e.g., music and games) or physical goods (e.g., books and consumer electronics) (Kim et al., 2010). It is considered a potential “killer” of cash, bank cards, and even Internet payments because it can be used to perform transactions in both a remote online store and a physical store (Ondrus et al., 2009).

Scholars have identified four points of convenience for consumers who use mobile payment (Boden et al., 2020). First, mobile payment eliminates spatial constraints on payment, enabling consumers to purchase products from a remote online shop (Slade et al., 2015). Second, mobile payment allows consumers to pay for their goods without the constraints of requiring a physical wallet (Mallat, 2007), cash, or credit card (Pham and Ho, 2015). Third, mobile payment has some advantages in terms of economic transaction performance characteristics (e.g., speed) compared with traditional payment tools (Teo et al., 2015). Fourth, mobile payment protects consumers from counterfeit money (Teo et al., 2015).

There are three main types of mobile payment tools as follows: short message service (SMS), near-field communication (NFC), and QR codes. SMS mobile payments are remote systems that require a communication protocol enabling the exchange of short text messages between two mobile devices (Valcourt et al., 2005). Both NFC and QR codes are proximity systems. NFC payment is based on radio frequency channel communication connecting payment devices and vending terminals without depending on mobile networks (Coskun et al., 2013). Although NFC payments are used in many scenarios, such as public transportation, their popularity is limited by the lack of devices due to the relatively high cost of NFC modules. The China UnionPay survey reported that the usage rate of NFC mobile payments was only 8% of all mobile payments in 2020 (CUP, 2021).

A QR code is a storage system employing a dot matrix or two-dimensional bar code that can be printed on study or shown on a screen to provide information and is recognized by special devices. As a payment solution, the QR code enjoys both low cost and high popularity among customers and businesses. In fact, QR code mobile payment has the potential to replace cash and credit cards in the physical store. For example, consumers can use the camera of their mobile phone to scan the QR code of the supermarket at the checkout to pay for goods. Alternatively, consumers can show a QR code on the screen of their mobile phone to a store employee, who can then scan the code using a scanner or their own mobile phone. The QR code is linked to the credit or debit card of the client. If the client does not have sufficient funds to make the QR code payment, the store will submit the payment claim later. The most popular use of QR code payments in China is through third-party payment platforms such as Alipay and WeChat Pay, rather than *via* mobile client apps provided by banks.

### Determinants of Mobile Payment Adoption

The extant literature on mobile payment focuses primarily on its utilitarian function from the perspective of technological system adoption. For instance, based on the technology acceptance model (TAM) theory, Kim et al. (2010) created a framework that includes the user-specific characteristics (personal innovativeness and mobile payment knowledge) and mobile payment system characteristics (mobility, reachability, compatibility, and convenience) to test the perceived ease of use on the intention to use mobile payment. Similarly, Yan et al. (2021) demonstrated the impact of transaction factors, perceived usefulness, and personal factors on the adoption of QR code mobile payment.

Applying an extended Unified Theory of Acceptance and Use of Technology (UTAUT) model, Slade et al. (2015) explored the antecedents (such as performance expectancy, social influence, innovativeness, and perceived risk) that significantly influenced behavioral intentions of non-users to use mobile payment in the United Kingdom. Similarly, Oliveira et al. (2016) revealed that compatibility, perceived technology security, performance expectations, innovativeness, and social influence directly and indirectly affected the adoption of mobile payment in Portugal.

de Kerviler et al. (2016) explored the benefits (utility, hedonic, and social) and risks (financial and privacy-related) of in-store mobile payment. Teo et al. (2015) discovered that effort expectancy (EE) and facilitating conditions (FC) positively affected behavioral intention to use mobile payment. Park et al. (2019) investigated multiple benefits (convenience, economy, enjoyment, and experiential) that positively influenced attitudes and adoption toward mobile payment among consumers in the United States.

### COVID-19 and Quick Response Code Mobile Payment

The year 2020 witnessed the accelerated popularization of QR codes in response to the COVID-19 pandemic. Health QR codes in China are officially authorized electronic certificates of personal health status; they have been used for contact tracing, exposure risk self-triage, self-updating of health status, healthcare appointments, and contact-free psychiatric consultations to curb the spread of COVID-19 (Nakamoto et al., 2020). When entering public places (e.g., public transportation systems, malls, offices, institutions, and schools), individuals are required to present their health QR code. Health QR codes have played an important role in the containment of COVID-19.

In addition, the ubiquity of health QR codes has spawned the spontaneous use of QR codes for mobile payment due to the demand for socially distanced, contact-free shopping. To register for mobile payment using QR codes, individuals must connect their ID card and bank card to a third-party app (e.g., WeChat and Alipay) and complete an application. Previously, many potential users hesitated to apply for QR code mobile payment due to privacy concerns, financial security considerations, and the process being cumbersome. Consequently, it is worthwhile to discuss how and why many people have changed their minds and adopted QR code mobile payment during COVID-19.

Using QR codes to pay for purchases is not common everywhere. Although mobile payments are more convenient than traditional methods such as cash or bank cards, the adoption rate of QR code mobile payment is still very low in many countries; in Malaysia, for example, only 10% of all mobile payments are made using a QR code (Yan et al., 2021). Although cashless transactions may be the way of the future, the adoption growth rate of mobile payment *via* QR codes is still lower than expected. While transaction convenience and transaction speed have been found to be important utility antecedents for QR code mobile payment usage (Yan et al., 2021), personal factors (e.g., optimism and innovativeness) should not be ignored.

### Social Learning Theory

Social learning theory provides a structured approach to understand how individuals learn information or behaviors by observing the environment and imitating others (Bandura, 1978). Social learning theory posits a two-interaction model to explain the phenomenon of social learning. Learners experience both external interactions with their social, cultural, and material environment and an internal psychological process with mental elaboration (Illeris, 2003). Emphasizing social participation, social communication, and social cooperation, the external interaction process generates benefits that stem from society. The internal psychological process includes the cognitive appraisal, which represents the understanding and ability to construct the meaning of learners. Based on beliefs and knowledge, cognitive appraisals are evaluations of the utility of specific actions to support the decision of whether to imitate those actions (Heijden, 2004; Lee et al., 2012).

Previous studies have shown that social learning theory can be applied to various economic and social behaviors. People are used to learning from others, especially those with whom they are familiar. In the social-commercial scenario, consumers acquire knowledge from their interaction with online communities, reviews, and recommendations (Huang and Benyoucef, 2013). Based on external interaction and the internal psychological process, consumers acquire enough knowledge and experience to support their purchase decisions (Chen et al., 2017b). Consumers also collect information through social media to assist with the cognitive appraisal before making a purchase (Hajli, 2012).

In fact, external stimuli from the environment play an important role in the adoption of QR code mobile payment. For instance, the COVID-19 pandemic has changed the purchase behaviors of consumers, such as by forcing them to maintain a safe distance from others in physical stores, minimizing direct physical contact with products or people, and using contactless payment methods rather than cash or credit cards. Since 2020, Chinese consumers have increasingly adopted QR code payment methods in order to satisfy the need for social distancing in public places. Both the health QR code and payment QR code are based on two social media apps (WeChat Pay and Alipay); therefore, it is convenient for new users to transfer their experience of the health QR code to the payment QR code. Moreover, the influence of other people is the strongest predictor of mobile payment adoption (Slade et al., 2015). Specifically, the positive opinions of family members, relatives, friends, and superiors can encourage individuals to adopt mobile payment (Oliveira et al., 2016). Thus, social learning theory is appropriate to explain the adoption of QR code mobile payment.

### Perceived Benefit

Perceived benefit is defined as the degree to which consumers believe they will benefit from adopting technology systems (Ng et al., 2009). Perceived benefit is affected by external variables; for example, social influence has been shown to be positively related to mobile payment benefits (Slade et al., 2015). Utilitarian benefit refers to the functional value resulting from using a system (Venkatesh and Brown, 2001). The utilitarian benefit of mobile payment has attracted extensive attention. For instance, de Kerviler et al. (2016) identified convenience as the main benefit of in-store mobile payment, while Yan et al. (2021) claimed that transaction speed and convenience were the main benefits offered by mobile payment.

According to the health belief model, the perceived health benefit is defined as the perceived effectiveness of protective actions in reducing health risks (Janz and Becker, 1984). The perceived health benefit is widely used to evaluate food consumption, medication regimens, and computer security behavior (Ng et al., 2009). When hearing or reading about the pandemic on the news and observing actions of others in relation to mobile payment, positive appraisal of individuals (such as perceived health benefit) on mobile payment may be enhanced.

## Hypothesis Development

**FIGURE 1:**
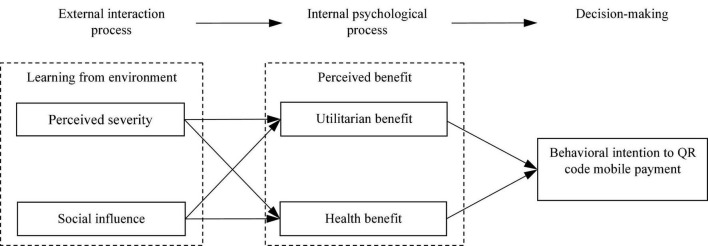
Research model.

According to social learning theory, two processes combine to form social learning: external interaction and an internal psychological process (Illeris, 2003). In the context of QR code mobile payment, the learning behavior of consumers in the interaction with other people is the external interaction process. Specifically, perceived severity and social influence were the stimuli from the external environment during the COVID-19 pandemic. Furthermore, the perception of utilitarian and health benefits to be gained from QR code mobile payment represents the internal psychological process. The research model of this study, based on social learning theory, is shown in Figure 1.

According to the literature on social learning, various information technology adoption behaviors derive from learning from others. Consumers can observe and replicate the behaviors of other people. For instance, empirical evidence shows that learning from social commerce components (e.g., forums, communities, ratings, reviews, and recommendations) generates positive attitudes (Chen et al., 2017a,b) and diminishes demand uncertainty (Chen et al., 2017a) toward a social commerce website. Similarly, social learning theory can be employed to understand the diffusion process of an enterprise application in an organization (Lorenzo et al., 2012).

Human beings adapt their behavior to respond to threats from the external environment. Perceived severity is defined as the perceived seriousness of a potential health problem (Ng et al., 2009). People were able to learn about the severity of COVID-19 through interaction with the surrounding environment, including the media, other people, and the health QR code issued by the Chinese government (Nakamoto et al., 2020). Based on protection motivation theory (PMT), individuals evaluate the benefits of protective behavior based on their ability to judge severity (Bockarjova and Steg, 2014). Accordingly, during the COVID-19 pandemic, people attempted to regulate their consumption behavior in order to avoid infection. For example, people who perceived the severity of the COVID-19 pandemic to be high undertook more extensive behavioral changes (Fragkaki et al., 2021). In particular, the perceived severity of the COVID-19 pandemic positively relates to attitudes and behaviors geared toward protection (Prasetyo et al., 2020). In addition, mobile payment is a useful tool with both utilitarian and health benefits; it was also officially recommended by WHO (2020) during the COVID-19 pandemic. Therefore, we inferred that the perceived severity of COVID-19 may have stimulated a positive appraisal of the utilitarian and health benefits of QR code mobile payment.

Based on the above considerations, we posited the following hypotheses:

H1. The perceived severity of COVID-19 positively affected the perceived utilitarian benefit of QR code mobile payment.

H2. The perceived severity of COVID-19 positively affected the perceived health benefit of QR code mobile payment.

Social influence from others affects health-related behaviors. For instance, social influence factors have been proven to be predictors of the acceptance of healthcare information technology in hospitals (Lu et al., 2020). Social support was found to be a key trigger of value co-creation behavior in online health communities (Liu et al., 2020). Thus, in the context of COVID-19, opinions from important others (e.g., family, friends, and superiors at work) can be powerful motivators to accept contactless methods of payment. In fact, according to the theory of social influence, support from important others may encourage adoption of a particular technology by consumers (Venkatesh et al., 2012). Learning from usage and/or recommendations of others, consumers acquire enough information to understand the utility or risk of a new technology. For example, Slade et al. (2015) determined that social influence was the strongest predictor of behavioral intention toward mobile payment in the United Kingdom. An online survey from Amazon’s Mechanical Turk (MTurk) also showed that social influence positively affected the perceived benefits (e.g., convenience, economic, informational, enjoyment, experiential, and social benefits) of mobile payment of consumers (Park et al., 2019).

In view of the health risk posed by COVID-19, the utility of mobile payment is not limited to efficiency and transaction speed; it also offers improved safety by reducing physical contact and increasing social distancing, which, in turn, help to contain the spread of COVID-19 (Yan et al., 2021). Thus, in the internal psychological process of social learning, we suggested the inclusion of health benefits in addition to utilitarian benefits. Based on the above literature and discussion, we hypothesized that social influence positively affected the internal psychological process in favor of the adoption of QR code mobile payment during the COVID-19 pandemic:

H3. Social influence positively affected the perceived utilitarian benefit of QR code mobile payment during the COVID-19 pandemic.

H4. Social influence positively affected the perceived health benefit of QR code mobile payment during the COVID-19 pandemic.

Published studies have explained how technology adoption results from perceived benefit. For example, utilitarian benefits (e.g., convenience and transaction speed) have been shown to be the main considerations when adopting mobile payment (de Kerviler et al., 2016). In addition, the perceived health benefit refers to the perceived effectiveness of specific behaviors in decreasing health risks (Janz and Becker, 1984). The perceived health benefit is an important factor in assessing specific behaviors such as food consumption, medication regimens, and computer security behavior (Ng et al., 2009). Based on the evidence outlined above, we inferred that both utilitarian and health perceived benefits may have influenced the adoption of QR code mobile payment during the COVID-19 pandemic. Therefore, we posited the following hypotheses:

H5. The perceived utilitarian benefit positively affected the intention to adopt QR code mobile payment during the COVID-19 pandemic.

H6. The perceived health benefit positively affected the intention to adopt mobile payment during the COVID-19 pandemic.

## Research Methodology

### Measurement

This study uses a survey questionnaire to collect data. The questionnaire items measuring each construct were adapted from extant influential literature to suit the context of mobile payment. We adopted “social influence” from Slade et al. (2015) to measure the impact of important others on the usage of QR code mobile payment. We further adopted “perceived severity” from Shafiei and Maleksaeidi (2020) to measure learning about the pandemic, “utilitarian benefit” from Chin et al. (2003) to measure the functionality of mobile payment in a transaction, “health benefit” from Hojjati et al. (2015) to measure health belief with respect to mobile payment, and “behavioral intention” from Yan et al. (2021) to measure attitude to mobile payment. Respondents were asked to rate the extent of their agreement on a seven-point scale (1 = strongly disagree, 7 = strongly agree) for the three items in each of these sections of the questionnaire, which comprised a total of 15 items. The measurement items and their sources in the literature are summarized in Table 1.

**TABLE 1:** Measurement items.

Construct	Measurement items	Sources
Perceived severity (PS)	PS1: COVID-19 has become a serious threat for humankind.	Shafiei and Maleksaeidi (2020)
	PS2: The negative impacts of COVID-19 are severe.	
	PS3: The news on COVID-19 scares me.	
Social influence (SI)	SI1: People who are important to me think that I should use mobile payment.	Slade et al. (2015)
	SI2: People who influence my behavior think that I should use mobile payment.	
	SI3: People whose opinions I value prefer that I use mobile payment.	
Utilitarian benefit (UB)	UB1: Using QR code mobile payment can make my life easier.	Chin et al. (2003)
	UB2: QR code mobile payment is useful.	
	UB3: I can benefit from using QR code mobile payment.	
Perceived benefit (PB)	PB1: Using mobile payment will protect my health from COVID-19.	Hojjati et al. (2015)
	PB2: Using mobile payment will help me avoid becoming infected with COVID-19.	
	PB3: Using mobile payment decreases my risk of getting infected with COVID-19.	
Behavioral intention (BI)	BI1: I intend to increase my use of QR code mobile payment in the future.	Yan et al. (2021)
	BI2: I intend to use QR code mobile payment when the opportunity arises.	
	BI3: I would like to use QR code mobile payment for purchasing instead of traditional payment methods.	

### Sample and Data Collection

We administered the survey online through professional research and modeling integrated data platform called Credamo^1^ from March 17 to April 16, 2021. As shown in Table 2, the sample comprised more male (59.3%) than female (40.7%) respondents. Most respondents (65.32%) were between 21 and 30 years old. In terms of educational level, most respondents had a bachelor’s degree as their highest educational attainment (80.2%). In terms of occupation, more than half of the respondents were employed by corporations (56.9%). Furthermore, nearly half of the respondents (45.56%) earned a monthly income in the range of CNY 5,001–9,000, and nearly half (49.6%) spent 1–3 h/day on social networking services (SNS) such as WeChat. Finally, most respondents (71.3%) made QR code mobile payments more than 21 times per month during 2020.

**TABLE 2:** Demographic information of respondents (*n* = 248).

Characteristics		Frequency	Percentage
Gender	Male	147	59.3
	Female	101	40.7
Age (years)	<21	19	7.66
	21–30	162	65.32
	31–40	62	25
	41–50	4	1.61
	>50	1	0.4
Education	Middle school	4	1.6
	High school	9	3.6
	Bachelor’s degree	199	80.2
	Master’s degree	31	12.5
	Ph.D.	5	2
Occupation	Government	3	1.2
	Public institution	50	20.2
	Corporate	141	56.9
	Student	42	16.9
	Freelancer or self-employed	12	4.8
Personal income per month	≤ CNY 1000	10	4.03
	CNY 1001–5000	75	30.24
	CNY 5001–9000	113	45.56
	≥ CNY 9001	50	20.16
Length of SNS usage per day	<1 h	6	2.42
	1–3 h	123	49.6
	4–6 h	91	36.69
	>6 h	28	11.29
Frequency of QR code mobile payment usage per month in 2020	1–20 times	71	28.7
	21–40 times	82	33.0
	>41 times	95	38.3

## Statistical Analysis

We employed the PLS-SEM method *via* SmartPLS 3.2.8 to test the proposed research model. PLS-SEM is a suitable method with high prediction accuracy in complex research models.

### Common Method Bias

Collecting multiple variable data from the same respondents *via* a questionnaire may incur common method bias (CMB). We adopted two methods to check the extent of CMB. First, we employed Harman’s single factor test to evaluate CMB. Through the assessment of unrotated exploratory factor analysis, the largest factor explained 35.146% of the total variance without any single factor explaining most of the covariance of the variables. Therefore, we concluded that our statistical results were unlikely to have been affected by CMB. Second, we used a common method factor analysis to check the severity of CMB (Liang et al., 2007). Results tabulated in Table 3 show that all substantive factor loadings (*R*_a_) were statistically significant at *p* < 0.001, with an average of 0.824. However, 13 of the 15 method factor loadings (*R*_b_) were not significant, with an average of −0.002. Therefore, CMB did not significantly affect the proposed model in this research.

**TABLE 3:** Common method factor analysis.

Construct	Indicator	Substantive factor loading (R_a_)	Sig.	R_a_^2^	Method factor loading (R_b_)	Sig.	R_b_^2^
Perceived severity (PS)	PS1	0.826	***	0.854	0.059	NS	0.003
	PS2	0.841	***	0.864	−0.006	NS	0.000
	PS3	0.777	***	0.822	−0.066	NS	0.004
Social influence (SI)	SI1	0.835	***	0.860	0.052	NS	0.003
	SI2	0.904	***	0.913	−0.042	NS	0.002
	SI3	0.861	***	0.879	−0.011	NS	0.000
Utilitarian benefit (UB)	UB1	0.8	***	0.837	0.038	NS	0.001
	UB2	0.799	***	0.836	−0.043	NS	0.002
	UB3	0.788	***	0.829	−0.001	NS	0.000
Health benefit (HB)	HB1	0.852	***	0.872	0.015	NS	0.000
	HB2	0.903	***	0.912	−0.086	NS	0.007
	HB3	0.798	***	0.835	0.068	NS	0.005
Behavioral intention (BI)	BI1	0.665	***	0.762	0.153	*	0.023
	BI2	0.83	***	0.857	−0.001	NS	0.000
	BI3	0.884	***	0.897	−0.163	*	0.027
Average		0.824		0.855	−0.002		0.005

***p < 0.001, **p < 0.01, *p < 0.05, NS p > 0.05.

### Measurement Model Evaluation

We evaluated the convergent validity and discriminant validity of the measurement model. In regard to the convergent validity, we checked Cronbach’s alpha, composite reliability (CR), the factor loading of indicators, and the average variance extracted (AVE). The results, shown in Table 4, reveal that factor loading of most indicators was above 0.7, Cronbach’s alpha values of all constructs were above the threshold of 0.7, CR of all constructs was more than 0.8, and AVE of all constructs was more than 0.5. These results imply that all constructs in the research model have good convergent validity (Hair et al., 2014). We further tested the discriminant validity (Hair et al., 2017) by comparing the AVE square root of each construct with its correlation coefficients. The results, shown in Table 5, reveal that the AVE square roots of all constructs are higher than their correlations, indicating that the constructs of the research model have sufficient discriminant validity.

**TABLE 4:** Results of convergent validity tests.

Construct	Item	Loading	Cronbach’s alpha	Composite reliability (CR)	Average variance extracted (AVE)	rho_A
Perceived severity (PS)	PS1	0.884	0.745	0.853	0.661	0.788
	PS2	0.844				
	PS3	0.701				
Social influence (SI)	SI1	0.881	0.835	0.901	0.751	0.839
	SI2	0.868				
	SI3	0.851				
Utilitarian benefit (UB)	UB1	0.827	0.708	0.837	0.632	0.712
	UB2	0.757				
	UB3	0.799				
Health benefit (HB)	HB1	0.864	0.809	0.887	0.724	0.811
	HB2	0.834				
	HB3	0.854				
Behavioral intention (BI)	BI1	0.803	0.7	0.833	0.625	0.709
	BI2	0.83				
	BI3	0.736				

**TABLE 5:** Discriminant validity (Fornell and Larcker criterion).

	PS	SI	UB	HB	BI
PS	***0.813***				
SI	0.263	***0.867***			
UB	0.426	0.394	***0.795***		
HB	0.24	0.431	0.378	***0.851***	
BI	0.347	0.398	0.543	0.544	***0.791***

*The diagonal elements in bold and italics are the average variance extracted (AVE) square roots of the constructs, while the off-diagonal elements are the inter-construct correlations.*

### Structural Model Evaluation

We employed a bootstrapping test with 5,000 resamples to estimate each hypothesis and its significance. The results indicate the support for all six hypotheses (Table 6). PS (β = 0.346, *p* < 0.001) and SI (β = 0.303, *p* < 0.001) were significantly related with UB; PS (β = 0.136, *p* < 0.05) and SI (β = 0.395, *p* < 0.001) were significantly related with HB. Furthermore, UB (β = 0.394, *p* < 0.001) and HB (β = 0.395, *p* < 0.001) were significantly associated with BI to adopt QR code mobile payment. In the research model, 42.8% of the variance in BI was explained by other variables, and this variance was found to be substantial.

**TABLE 6:** Outcome of the structural model.

Path	β	Significance	Result	Effect size (*f*^2^)	*R* ^2^	Predictive relevance (*Q*^2^)
PS→UB	0.346	***	H1 supported	0.152	0.267	0.155
SI→UB	0.303	***	H3 supported	0.117		
PS→HB	0.136	*	H2 supported	0.022	0.203	0.136
SI→HB	0.395	***	H4 supported	0.182		
UB→BI	0.394	***	H5 supported	0.232	0.428	0.251
HB→BI	0.395	***	H6 supported	0.234		

***p < 0.001, *p < 0.05.

Additionally, results in Table 6 show that our research model can explain 26.7% of the variance in utilitarian benefit (UB, *R*^2^ = 0.267) of, 20.3% of the variance in health benefit (HB, *R*^2^ = 0.203) of, and 42.8% of the variance in behavioral intention (BI, *R*^2^ = 0.428) to adopt QR code mobile payment. Moreover, Table 6 shows that all the effect size values (*f*^2^) range between 0.02 and 0.35, and that all the predictive relevance values (*Q*^2^) are above zero, confirming the high predictive relevancy of the research model.

## Discussion

This study examines the QR code mobile payment adoption process in China during the COVID-19 pandemic, from the perspective of social learning theory. QR code mobile payment provides consumers with higher transaction efficiency and a contactless in-store experience, which decreases the risk of infection. This study investigates how external interaction and the internal psychological process affect decision-making about QR code mobile payment usage. The empirical results reveal that perceived severity and social influence are positively related to utilitarian and health benefits, supporting hypotheses H1–H4. Utilitarian and health benefits are shown to be positively associated with behavioral intention to adopt QR code mobile payment, supporting hypotheses H5 and H6.

### Theoretical Contribution

This study contributes to the current literature in several ways. First, we proposed a theoretical model to explore the intention of customers to use QR code mobile payment during the COVID-19 pandemic, augmenting the understanding of mobile payment adoption behavior in physical stores during the pandemic. Besides posing a major threat to human safety, the pandemic has affected everyday consumption behaviors. Although consumers have become increasingly apprehensive about interpersonal contact in physical stores, research on the methods they use to protect themselves in shopping areas is lacking. This study sheds light on how learning from the external environment affects the usage of QR code mobile payment of consumers. The research model provides novel insights about the external interaction and psychological processes involved in the adoption of mobile payment, thus offering a new avenue for future research on consumer behavior.

Second, this study broadens our understanding of the benefit appraisal process with regard to mobile payment usage. Although prior research has focused on utilitarian benefits such as transaction speed and convenience (Yan et al., 2021), the proposed model in this study suggests that the perceived health benefit may also enhance the behavioral intention to use QR code mobile payment. This discovery adds to the literature on the benefit appraisal process and encourages further research examining the importance of the health utility provided by contactless payment tools. These findings also respond to calls from past studies (Yan et al., 2021).

Third, the research model has important implications for social learning theory. Previous research has applied this theory in the areas of enterprise application (Lorenzo et al., 2012), social commerce (Chen et al., 2017a,b), and virtual community (Chou, 2020). This study augments the literature on social learning theory by exploring the external interaction and internal psychological process associated with adopting QR code mobile payment during the COVID-19 pandemic.

### Managerial Implications

The findings in this study have several managerial implications for the extension of QR code mobile payment. They shed light on the adoption of QR code mobile payment of consumers under the influence of external interactions during the COVID-19 pandemic. The external impact of perceived severity and social influence triggers the internal psychological process of appraising QR code mobile payment, which implies that the threat of environmental risks such as COVID-19 can be important antecedents to the uptake of mobile payment. First, our findings suggest that companies can encourage the use of QR code mobile payment by reminding consumers of the pandemic. For instance, physical stores could telecast news about COVID-19 worldwide to stimulate the appraisal of, and intention to adopt, mobile payment of consumers. Second, this study suggests that companies could include the health benefit of reducing interpersonal contact as an advantage when promoting QR code mobile payment. For instance, companies or stores could persuade consumers to use mobile payment by labeling it a “smart choice for your health.”

### Limitations and Future Study

Although rigorous theoretical and methodological procedures were applied in our research model, this study is not without limitations. First, the model only concentrates on the utilitarian and health benefits in the internal appraisal process, without considering other factors such as self-efficacy, innovativeness, and trust. Such factors may provide more detailed insights into the usage of QR code mobile payment. Second, the sample size of this study constrains the generalizability of the research results. Thus, in future studies, large-scale, cross-national surveys could be conducted to confirm the findings of this study. Third, this study only investigates the behavioral intention to use QR code mobile payment on the part of consumers, without considering the attitudes of shopkeepers. Therefore, we recommend that future studies also explore the factors affecting usage intention from the perspective of the retailer.

## Data Availability Statement

The original contributions presented in the study are included in the article/supplementary material, further inquiries can be directed to the corresponding author.

## Ethics Statement

The studies involving human participants were reviewed and approved by Ethics Committee of Huazhong Agricultural University. Written informed consent for participation was not required for this study in accordance with the national legislation and the institutional requirements.

## Author Contributions

MT: conceptualization, methodology, and writing—original draft preparation, and review and editing. LW and HW: writing—review and editing and funding acquisition. ZD and ZG: data collection and editing. JC: data collecting, data analysis, revising, and final approval.

## Conflict of Interest

The authors declare that the research was conducted in the absence of any commercial or financial relationships that could be construed as a potential conflict of interest.

## Publisher’s Note

All claims expressed in this article are solely those of the authors and do not necessarily represent those of their affiliated organizations, or those of the publisher, the editors and the reviewers. Any product that may be evaluated in this article, or claim that may be made by its manufacturer, is not guaranteed or endorsed by the publisher.

## Footnotes

1
www.credamo.com
